# Prevalence of malaria and associated risk factors among household members in South Ethiopia: a multi-site cross-sectional study

**DOI:** 10.1186/s12936-024-04965-4

**Published:** 2024-05-12

**Authors:** Girma Yutura, Fekadu Massebo, Nigatu Eligo, Abena Kochora, Teklu Wegayehu

**Affiliations:** 1 Malaria and Neglected Tropical Diseases, Gorche Health District, Hawassa, Sidama Regional State Ethiopia; 2 Department of Biology, College of Natural and Competitional Sciences, Arba Minch University, Arba Minch, Ethiopia; 3 Malaria and Neglected Tropical Diseases, Armauer Hansen Research Institute, Addis Ababa, Ethiopia

**Keywords:** Febrile, Malaria, *Plasmodium*, Risk factors, South Ethiopia

## Abstract

**Background:**

Despite continuous prevention and control strategies in place, malaria remains a major public health problem in sub-Saharan Africa including Ethiopia. Moreover, prevalence of malaria differs in different geographical settings and epidemiological data were inadequate to assure disease status in the study area. This study was aimed to determine the prevalence of malaria and associated risk factors in selected rural *kebeles* in South Ethiopia.

**Methods:**

A community-based cross-sectional study was conducted between February to June 2019 in eight malaria-endemic *kebeles* situated in four zones in South Ethiopia. Mult-stage sampling techniques were employed to select the study zones, districts, *kebeles* and households*.* Blood sample were collected from 1674 participants in 345 households by finger prick and smears were examined by microscopy. Sociodemographic data as well as risk factors for *Plasmodium* infection were collected using questionnaires. Bivariate and multivariate logistic regressions were used to analyse the data.

**Results:**

The overall prevalence of malaria in the study localities was 4.5% (76/1674). The prevalence was varied among the study localities with high prevalence in Bashilo (14.6%; 33/226) followed by Mehal Korga (12.1%; 26/214). *Plasmodium falciparum* was the dominant parasite accounted for 65.8% (50/76), while *Plasmodium vivax* accounted 18.4% (14/76). Co-infection of *P. falciparum* and *P. vivax* was 15.8% (12/76). Among the three age groups prevalence was 7.8% (27/346) in age less than 5 years and 7.5% (40/531) in 5–14 years. The age groups > 14years were less likely infected with *Plasmodium* parasite (AOR = 0.14, 95% CI 0.02–0.82) than under five children. Non-febrile individuals 1638 (97.8%) were more likely to had *Plasmodium* infection (AOR = 28.4, 95% CI 011.4–70.6) than febrile 36 (2.2%). Individuals living proximity to mosquito breeding sites have higher *Plasmodium* infection (AOR = 6.17, 95% CI 2.66–14.3) than those at distant of breeding sites.

**Conclusions:**

Malaria remains a public health problem in the study localities. Thus, malaria prevention and control strategies targeting children, non-febrile cases and individuals living proximity to breeding sites are crucial to reduce malaria related morbidity and mortality.

## Background

Malaria continues to remain a global burden and a public health threat despite increasing efforts aimed at improving vector control, therapeutics and diagnostics approaches worldwide [1]. According to World Health Organization (WHO), there were 249 million estimated malaria cases in 85 malaria endemic countries in 2022, an increase of 5 million cases compared with 2021 [1]. Most of the increase in case numbers and deaths over the past 5 years occurred in countries in the WHO African Region. Ethiopia is one of the main countries contributing to the increase in cases and death between 2021 and 2022 [1].

In Ethiopia, malaria transmission is seasonal with two peak transmissions seasons following the bimodal rainfall pattern. Like in most parts of Ethiopia, the peak season for the transmission of malaria in the current study area is from September to December, following the major rainy season [2]. It affects two-thirds of landmass with 60% of the population living in low to high malaria risk areas, making malaria a leading public health problem in the country [3]. *Plasmodium falciparum* and *Plasmodium vivax* accounting to 60% and 40% of the disease in the country [2, 4]. *Plasmodium falciparum* is highly virulent species which causes severe malaria and death in the country [5, 6]. In the country, there were 2.78 million cases and 8041 deaths were reported in 2021 [7].

Ethiopia is currently working on a malaria elimination programme that aims to eradicate the disease by 2030 [8, 9]. In the fight against the disease, the distribution of long-lasting insecticidal nets (LLINs) and indoor residual spraying (IRS) are critical. Additionally, increased healthcare utilization, early diagnosis, prompt treatment, prevention, and rapid management of malaria epidemics, were among the interventions used. However, malaria control programmes need to target active case detection for capturing asymptomatic infections as it challenges the ongoing malaria control and elimination efforts worldwide [10, 11]. Most *P. falciparum* and *P. vivax* infections are likely to be asymptomatic [12]. Such infections are missed by passive surveillance, but remain infectious to mosquitoes. Treatment of asymptomatic carriers could help reduce disease transmission by depleting the reservoir of parasites available for infection of mosquitoes [13]. Without identification and targeting of asymptomatic infectious pool, transmission interruption might not be possible [12].

Several studies have been conducted to describe parasitological and entomological data of malaria in various malaria-endemic areas in Ethiopia. A recent study conducted in South Ethiopia has indicated *Anopheles arabiensis* to be the primary vector of *P. falciparum* after decades of malaria control [14]. On the other hand, studies consider malaria prevalence and risk in remote Ethiopian communities like the current study setting are limited. Therefore, a community-based study on malaria will provide data that is critical for making evidence-based decisions. The aim of the present study was to assess the prevalence of malaria and the associated risk factors among communities in various geographical settings in selected sites of South Ethiopia.

## Methods

### Study areas description

**Fig. 1:**
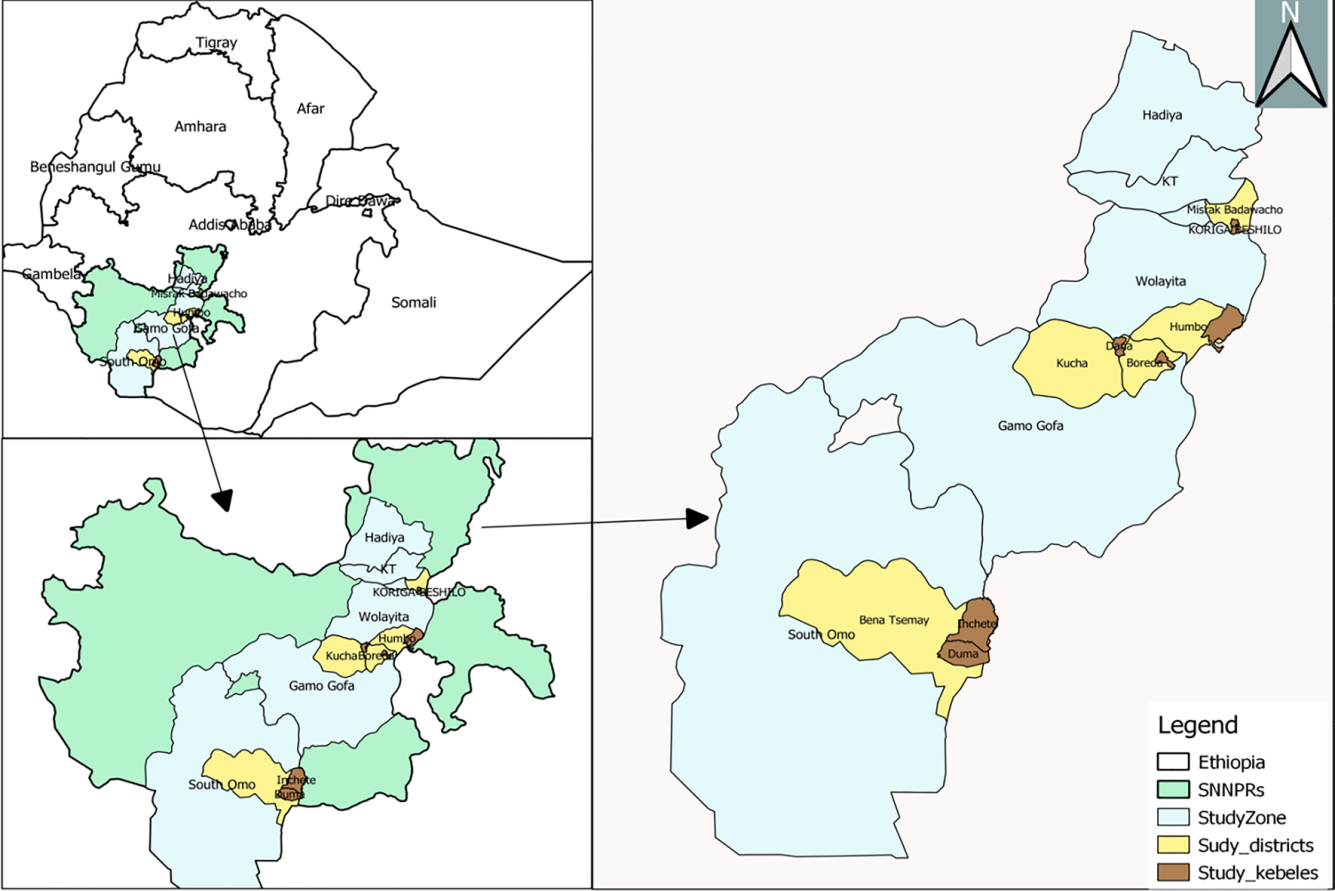
Map of study areas (Arc GIS version 10.1)

This study was conducted in four zones namely South Omo, Gamo, Wolaita, and Hadiya Zones of the former South Nations Nationalities Peoples Regional State (SNNPRs) (Fig. 1). The SNNPR was one of the regional states in Ethiopia, which include 17 administrative zones and 7 special *woredas*. The region has an elevation of 376 to 4207 m above sea level. Average elevation of the study *kebeles* ranged from 553 m a.s.l at Duma to 1720 m a.s.l. at Mehal Korga. The mean annual rainfall ranges from 500 – 2200 mm and temperature ranges between 15 °C and 30 °C. Malaria continues to be a significant health problem in the region, but the transmission intensity varies across different local settings [15].

### Study design and period

Community based cross-sectional study was conducted between February to June, 2019 to determine prevalence of *Plasmodium* infection and associated risk factors among household members in South Ethiopia.

### Study participants

People residing in all the study *kebeles* could be taken as source population and individuals in selected households were included as study participants based on the following inclusion and exclusion criteria.

### Inclusion and exclusion criteria

All household members who lived in the *kebele* for at least 6 months were included in the study regardless of the age and sex. Individuals, who receiving malaria treatment during survey and non-consenting respondents were excluded.

### Sample size determination and sampling techniques

The sample size was determined using single population proportion formula of Fink and Kosecoff [16] assuming, 16% expected prevalence [17], 2.5% margin error, design effect 2, α = 5% (95% confidence level), and 15% non-response rate. Accordingly, the sample size was calculated as follows:

$$n=\frac{(Z1-\frac{\alpha }{2}{)}^{2}*P*(1-P)}{{d}^{2}}$$

where n = the sample size, Z_1_-α/2 = the Z-value at a given confidence level, P = estimated prevalence of malaria in the study population, d = margin of error or sample error. Therefore, sample size was calculated as

$$\begin{aligned} n & = 1.96*1.96*0.16*0.74/0.025*0.025 \\ & = 728*15\% = 109,728 + 109 = 837, \\ & = 837*2 = 1674 \\ \end{aligned}$$

**Fig. 2:**
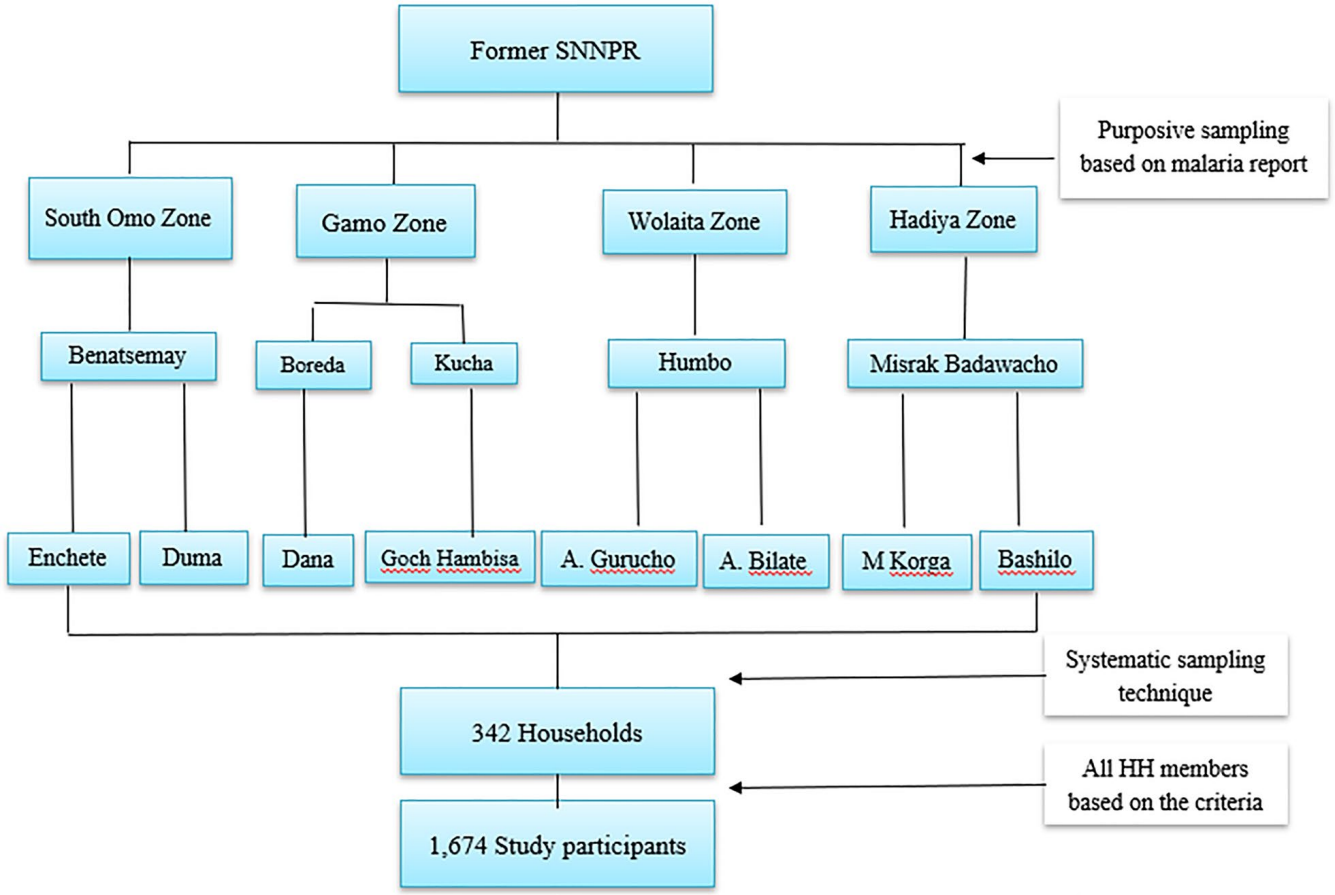
Sampling framework of the study sites and households

Multistage sampling was used to select districts, *kebeles*, and households. According to the zonal health department report, one high-malaria-prevalent district in each zone were included, except the Gamo zone, where two districts were included. The Gamo zone included two districts as it had wider geographical coverage during conception of the study as Gamo-Gofa Zone. However, the Gamo-Gofa Zone became two independent zones during the study period and two of the districts located in Gamo Zone. Finally, two malarious *kebeles* were purposefully selected in each district based on the malaria incidence (Fig. 2).

According to Ethiopian population and housing census of 2007, average family size for the region was 4.9 [18]; and hence the calculated household was 345 (Table 1). The total sample size (1674) was allocated to HHs proportionally to individual *kebeles* based on entire population of study sites as indicated in Table 1. Systematic sampling was carried out using the lists of households in each *kebele* health post to select the households. The first household was selected randomly by lottery method and every k^th^ household was included in the study. Where K is calculated by the formula of K = $\frac{N}{n}, {\text{K}}=\frac{4729}{345}$, Where, K = the gap between every household, N = total number of households in the study *kebeles* and n = sample size of households was calculated from individual sample size. Therefore, K = 13, thus every thirteenth household was included. Few houses were replaced by nearby houses when the selected household heads were absent or did not volunteer to participate in the study.

**Table 1:** Sample size allocation to study *kebeles* in four Zones in the South Ethiopia, February–June 2019

Zones	*Kebeles*	Total population	HH (Popl/4.9)	Sample size allocation	No. of sampled HHs
South Omo	Enchete	1934	395	140	29
	Duma	1607	328	116	24
Gamo	Dana	2980	608	216	44
	Gocho Hambisa	3232	660	234	49
Wolaita	Abaya Gurucho	3357	685	243	50
	Abaya Bilate	3351	684	243	50
Hadiya	Mehal Korga	3136	640	224	46
	Bashilo	3575	730	259	53
Total		23,172	4729	1674	345

HH (household) = total population/average family size (4.9), Sample size allocation = total population of individual *kebeles* * sample size (1674)/total population of all *kebeles* (23,172), Sampled HHs = Total HHs (4730) *individual allocated sample s/total Population (23,172)

### Sample collection and processing

#### Blood sample collection and processing

Capillary blood sample was collected using sterile blood lancets from participants after obtaining written consent during house-to-house visits. Blood sample collection was done by senior medical laboratory technicians, following standard guidelines [19]. Thick and thin blood smears were prepared at field and dried by air. The air dried blood thin and thick smears were transported to nearby health centres’ laboratories using slide boxes. The smears were fixed using 99.8% methanol, dried, and stained with a 10% Giemsa solution for 10 min. Then, microscopy was employed by experienced laboratory technicians to detect and identify *Plasmodium* parasite species according to laboratory guidelines. Slides were declared negative for *Plasmodium* parasites after thorough examination of 100 fields and no *Plasmodium* parasite is detected by microscopy.

#### Sociodemographic data collection

Sociodemographic data were collected from 345 households based on structured questionnaire. The questioner prepared in local language was sought information on sociodemographic characteristics, and malaria prevention and control practices. After having the written consents, both individual and household-level factors associated with malaria transmission was obtained from the participant. During the time of sample collection, fever of study participants was checked and signs and symptoms of malaria such as headache, chills, sweating were asked. Fever of individuals was measured using thermometers (Hanimax) and auxiliary body temperature (> 37.5 ℃) were considered as febrile.

### Data quality assurance

Data quality was maintained using various approaches. First, training was given for field assistants (data collectors) to have a common understanding to collect the appropriate demographic information. Second, blood sample collection and microscopy were done by senior laboratory technologists and discussion was held to apply standard operational diagnostic procedures during laboratory work. Each questioner and the collected sample were cross-checked for completeness, accuracy, and consistency by the group members and corrective measures taken. Moreover, all houses were coordinated using geographical position system and study individuals were coded during blood sample collection. All positive slides and 10% of negative slides were re-examined by another senior laboratory technologist blinded to previous slide results.

### Study variables

The outcome variable for examination of blood films was *Plasmodium* infection status.

Independent variables included house structure (the roof material, floor material, presence of visible holes on wall), IRS spraying in the last 12 months, LLINs ownership (presence of bed nets, total number of nets, access to LLINs and use of mosquito nets), presence of mosquito breeding site. The variables like sex, age, and fever (auxiliary temperature) were considered as individual level for analysis of data.

### Data analysis

Data was entered into Microsoft Excel spreadsheets and analysed using SPSS version 20.0. Descriptive statistics were used to determine the frequencies of variables. Bivariate logistic regression analysis was conducted to examine the association between *Plasmodium* infections with associated risk factors. Multivariate logistic regression analysis was conducted to test potential predicators’ variable that was the main risk factor for *Plasmodium* infection. The goodness of model fit was checked by Hosmer-Leme show-test and the logistic regression was fit for the test. Data normality was checked by non-parametric test of one-sample Kolmogorove-Smirnov test (1-sample K-S). Logistic regression statistical method of multivariate logistic regression was used with a 95% confidence interval and odds ratio was used to control confounders with the level of statistical significance was taken as *P*-value < 0.05 for analysis of independent and outcome variables. During binary logistic regression if the *p* ≤ 0.025 was considered as a candidate for multivariate logistic regression.

## Results

### Sociodemographic characteristics

The sociodemographic characteristic of the study participants was summarized in Table 2. From the total of 1674 participants, 748 (44.7%) were males and 926 (55.3%) were female. With regard to the age, 346 (20.7%), 531 (31.7%) and 797 (47.6%) were in the age groups < 5, 5–14 and > 14 age groups, respectively. Of the total, 1638 (97.8%) were non-febrile and the rest 36 (2.2%) were febrile cases.

**Table 2:** Sociodemographic characteristic of study participants by study *kebeles* in the South Ethiopia, February–June 2019

Characters		Frequency (n = 1674)	Percent
Sites	Abaya Gurucho	224	13.2
	Abaya Bilate	181	10.7
	Dana	272	16.0
	Gocho Hambisa	268	15.8
	Enchete	163	9.6
	Duma	126	7.4
	Mehal Korga	214	12.6
	Bashilo	226	13.3
Sex	Male	748	44.7
	Female	926	55.3
Age (years)	< 5	346	20.7
	5–14	531	31.7
	> 14	797	47.6
Febrile	Absence	1638	97.8
	Presence	36	2.2
LLINs utilization	Yes	1148	68.6
	No	526	31.4
IRS Spray	Yes	791	47.2
	No	883	52.8

### Overall, and site-specific prevalence of malaria

The overall prevalence of malaria was 4.5% (76/1674) confirmed by microscopy (Table 3). The *Plasmodium* infection was more prevalent in Bashilo *kebele* 14.6% (33/226) followed by Mehal Korga 12.1% (26/214). *Plasmodium* infection was detected in seven study *kebeles* and no malaria cases were detected in Gocho Hambisa *kebele*.

**Table 3:** Prevalence of *Plasmodium* species in eight *kebeles* of the South Ethiopia, February–June 2019

Zones	*Kebeles*	Total examined (N)	*Plasmodium* species			Total prevalence (%)
			*P. falciparum* n (%)	*P. vivax* n (%)	Mixed infection n (%)	
South Omo	Enchete	163	3 (1.8)	1 (0.6)	–	4 (2.5)
	Duma	126	1 (0.8)	–	–	1 (0.8)
Gamo	Dana	272	6 (2.2)	1 (0.4)	–	7 (2.6)
	Gocho Hambisa	268	–	–	–	–
Wolaita	Abaya Gurucho	224	1 (0.4)	1 (0.4)	–	2 (0.9)
	Abaya Bilate	181	1 (0.6)	2 (1.1)	–	3 (1.7)
Hadiya	Mehal Korga	214	15 (7.0)	8 (3.7)	3 (1.4)	26 (12.1)
	Bashilo	226	23 (10.2)	1(0.4)	9 (4.0)	33 (14.6)
Total (%)		1674	50 (3)	14 (0.8)	12 (0.7)	76 (4.5)

Among the confirmed malaria cases, *P. falciparum* was dominant species accounting 65.8% (50/76), while *P. vivax* was 18.4% (14/76). Mixed infections with *P. falciparum* and *P. vivax* were accounted 15.8% (12/76). Higher prevalence of *P. falciparum* 10.18% (23/226) was observed in Bashilo *kebele*. Among study *kebeles*, Mehal Korga had the high prevalence of *P. vivax* 3.74% (8/214) (Table 3).

### Sex and age-related prevalence of malaria

Of the study participants, 5.2% (39/748) males and 4% (37/926) females were found positive for *Plasmodium* parasite (Table 4). The prevalence of *Plasmodium* parasites among age groups were 7.8% (27/346) in under five children, 7.5% (40/531) in 5–14 years and 1.1 (9/797) in > 14 years. The greatest malaria prevalence was observed among under five children followed by school age groups.

**Table 4:** Sex and age-related prevalence of malaria in study setting of the South Ethiopia, February–June 2019

Variables		Study participants	Malaria positive cases	Percentage (%)
Sex	Male	748	39	5.2
	Female	926	37	4.0
Age	< 5	346	27	7.8
	5–14	531	40	7.5
	> 14	797	9	1.1

### Malaria-associated factors analysis

A total of eight independent variables were considered for bivariate logistic regression analysis of individuals and household associated risk factors for malaria parasite infections (Table 5). The variables associated with individual and household-level risk factors of malaria parasite infection was age, fever during survey time, LLINs utilization, IRS spray status, house structure (main roof material), main wall material, presence of visible hole on the wall, and living proximity to breeding sites. Among those variables, the age of individuals, fever, LLINs utilization and living proximity to the breeding site were a candidate for multivariate analysis.

**Table 5:** Binary and multivariate logistic regression analysis of associated factors for malaria in the study localities, in South Ethiopia, February–June 2019

Variables		Category		Binary logistic			Multivariate logistic		
		+ ve (%)	−ve (%)	P-value	COR	CI	P-value	AOR	CI
Age	< 5	27 (7.8)	319 (92.2)						
	5–14	40 (7.5)	491 (92.5)	0.89	0.97	0.58–1.60	0.83	1.08	0.54–2.14
	> 14	9 (1.1)	788 (98.9)	0.001*	0.14	0.07–0.31	0.029*	0.14	0.02–0.82
Fever	No	53 (3.2)	1585 (96.8)						
	Yes	23 (63.9)	13 (36.1)	0.001*	53.93	25.9–112.4	0.001*	28.4	11.4–70.6
LLINs Utilization	No	39 (7.4)	487 (92.8)						
	Yes	37 (3.2)	1111 (96.8)	0.001*	0.43	0.27–0.68	0.003*	0.37	0.19–0.72
IRS Spray	No	46 (5.2)	837 (94.8)	0.166	0.72	0.45–1.15	0.06	0.52	0.26–1.03
	Yes	30 (3.8)	761 (96.2)						
Roofing	Corrugated	28 (3.8)	710 (96.2)	0.195	1.37	0.85–2.21	0.23	0.66	0.33–1.31
	Thatched	48 (5.1)	888 (94.9)						
Wall	Wood	5 (1.4)	290 (98.6)	0.89	0.86	0.09–7.78	0.47	0.40	0.03–4.78
	Wood and mud	70 (5.4)	1246 (94.6)	0.21	3.53	0.48–25.8	0.39	2.46	0.31–19.3
	Wood cement	1 (1.6)	62 (8.4)	1					
Visible wall hole	No	42 (5.4)	733 (94.6)						
	Yes	34 (3.8)	861 (96.2)	0.12	0.69	0.43–1.10	0.63	0.85	0.44–1.64
Breeding site	No	12 (2.0)	578 (98.0)						
	Yes	64 (5.9)	1020 (94.1)	0.005*	2.6	1.34–5.05	0.001*	6.17	2.66–14.3

In the multivariate logistic regression analysis, the predictors of *Plasmodium* infections after controlling confounders of the variables were the age of individuals (AOR = 0.14, 95% CI 0.02–0.82) and fever during survey time (AOR = 0.37, 95% CI0.19–0.72). Household-level predictor variables of *Plasmodium* infections were LLINs utilization (AOR = 0.37, 95% CI 0.19–0.72) and proximity mosquito breeding sites (AOR = 6.17, 95% CI 2.66–14.3) were a significant association with *Plasmodium* infection.

The individuals who’s aged < 5 was 86% more likely to have a malaria as compared with individuals whose age > 14 with the p-value = 0.029 (IC = 0.02-0.82). Individuals who do not have a fever during study time were 28.4 times more likely have *Plasmodium* parasite as compared to individuals with fever with the p-value = 0.001 (CI 11.4–70.06).

LLINs utilization was significantly associated with *Plasmodium* species. The individuals that have not to use LLINs during a sleeping time were 63% more likely have a chance to *Plasmodium* parasite infection as compared with their counterparts with the p-value = 0.003 (CI 0.19–0.72) (Table 5). Those individuals who live proximity to the breeding site were 6.17 times more likely have a chance to develop malaria as compared to individuals do not live around breeding site with the p-value = 0.001 (CI 2.66–14.3).

## Discussion

Malaria affects the lives of almost all people living in sub-Saharan African countries. In Ethiopia, malaria remains a major public health problem despite continuous control and preventive strategies in place. The overall prevalence of malaria in this study was 4.5% with varying prevalence in different study sites in South Ethiopia. Both *P. falciparum* and *P. vivax* has been identified with *P. falciparum* dominant species accounted for 65.8% (50/76). It was also observed that lower age group, non-febrile case, and individuals who live proximity to mosquito breeding site had higher *Plasmodium* infection.

The overall prevalence of malaria in this study (4.5%) was in line with reports from various parts of Ethiopia including 4.4% in Butajira, 6.1% in Benatsemay district (South Omo), 6.7% in Dembia districts, 6.8% in Sanja town, and 4% in Jimma zone [20–23]. This finding is higher than the prevalence reported in another study in Butajira and national malaria indicator survey 2015 result, with prevalence of 0.9% and 0.5%, respectively [24, 25]. On the other hand, the present finding is much lower than the prevalence reported in Kisumu country in the Kenya with 28% [26], Armachiho districts, North West Ethiopia with 18.4% [27], and Dilla town and surrounding areas with 16.0% [17]. The difference in findings might be associated with sociodemographic, socioeconomic and environmental factors that could affect the epidemiology of malaria.

Prevalence of *Plasmodium* infection was relatively high in Bashilo (14.6%) and Mehal Korga *kebeles* (12.6%) as compared to Enchete, Duma, Dana, Gocho Hambisa, Abaya Gurucho and Abaya Bilate. The same holds true in other studies conducted in different parts of Ethiopia [20, 28, 29]. The heterogeneity of *Plasmodium* infection in the present study settings might be because of ecologic and environmental factors, host and vector characteristics, social, biological and socio demographic factors.

*Plasmodium falciparum* and *P. vivax* were identified as co-endemic species in study areas while *P. falciparum* was dominant species of parasite. The dominance of *P. falciparum* was consistent with the study conducted in Benatsemay districts in South Omo, Ethiopia [23]. In addition, the national community-based malaria indicator surveys conducted during peak malaria transmission season in the 2007 and 2011 reported the dominance of *P. falciparum* as 83% and 77%, respectively [30, 31]. The dominance of *P. falciparum* species might be more widely distributed in many parts of Ethiopia. This might be associated to the capacity of *P. falciparum* parasite to develop resistance against anti-malarial drugs represents a central challenge in the global control and elimination of malaria [32]. In contrast to this finding, other studies conducted in different geographical settings in Ethiopia [28, 29] monitoring changing of the epidemiology of malaria beyond Gark projects [33] and the facility-based cross-sectional study in Hadiya Zone [34] the *P. vivax* dominates over *P. falciparum*. One possible reason for predominance of *P. vivax* might be improper management of primaquine that lead to the relapse of hyponozoites.

Regarding the age groups, the likelihood of having higher malaria cases was found among under five children and school age children than other age groups. This finding was in line with malaria prevalence in Ethiopian on malaria indicator survey [25], in Arba Minch Zuria district [35] children this age groups are more vulnerable and had have *Plasmodium* parasite infections. The reason why high malaria cases in this age groups might be due to immunity status, more exposed to mosquito bites before bedding, and less awareness of self-care for utilization of malaria preventive measures.

Non-febrile *Plasmodium* infection was common in endemic areas. In malaria-endemic areas, people may develop partial immunity, allowing the non-febrile infection to occur. The odds of *Plasmodium* infection were higher in individuals that do not have fevers than those who have fever. The result consistent with the study conducted in Senegal that indicated *P. falciparum* was dominant species in asymptomatic cases [36]. In other way, in low transmission settings, asymptomatic cases are common and most of the asymptomatic infections are sub-microscopic [28, 37]. Study showed that asymptomatic cases could serve as reservoirs of infections to the mosquito vectors [38]. Thus, they could serve as a major source of gametocytes and contributed to residual transmissions of malaria as asymptomatic carriers do not visit health facility for treatment. In many countries *P. falciparum* is asymptomatic or sub-clinical. In very low transmission settings, sub-microscopic carriers may contribute up to 50% of humans to mosquito transmission [39].

Appropriate use of the utilization of LLINs is one of the key interventions for the prevention of malaria [40]. In this study, ownership of LLINs was 76.9%. This finding was higher than the previous findings in Hadiya zones with LLINs ownership of 41.6% [34]. On the other hand, national malaria indicator survey conducted in 2011 and 2015 showed 55% and 64% of households have at least one LLINs of any type [25, 30] and a community-based cohort study in South Central Ethiopia [41]. However, the accesses to LLINs were not significantly associated with *Plasmodium* infection in study sites.

The utilization of LLINs has an association with malaria cases among study participants. The current study showed that participants who use LLINs had lower malaria cases than those do not use. This findings is in line with the study conducted Dilla and surroundings areas, Dembia districts, and Hadiya zones where participants do not use bed nets were 0.2, 0.2 and 4.6 times more likely developed *Plasmodium* parasite infections, respectively [17, 22, 34]. The finding speculates the proper usage of LLINs protects from malaria through protecting mosquito bites depending on biting activity. It is noticeable that the proper utilization of LLINs will prevent mosquito that in turn prevent *Plasmodium* parasite infection. These findings might the implication of possession and efficacy of LLINs utilization in the community and less attention to frequent utilization in different local settings.

Another important factor that determines the odds of *Plasmodium* infection is living proximity to the breeding site. In this study, a participant who live proximity to mosquito breeding sites was at high risk of *Plasmodium* infections. The study participants those lives proximity to the stagnant water of mosquito the breeding sites 6.17 times more likely have a chance to develop *Plasmodium* infection as compared to individuals do not live around the breeding site. This finding in agreement with the study conducted in Dilla and surrounding areas and Dembia districts [17, 22] by increasing the probability of having *Plasmodium* infection. This is because proximity mosquito breeding sites give more chances to exposure mosquito bites in the community.

This study has some limitations. One of the limitations of this study is the laboratory diagnosis which is limited to microscopy only, a low sensitive tool. The second limitation is seasonality of transmission was not determined. The community-based nature of the study can be viewed as one of the strengths of this study as it enables us to screen the non-febrile cases who could serve as potential reservoir of malaria parasite. High response rate of study participants can also be viewed as another strength of this study.

## Conclusions

Malaria is still important public health problems, although the prevalence of disease was varying in the study sites*.* Lower age children, non-febrile cases and those who reside proximity to mosquito breeding sites were at higher risk of *Plasmodium* infection. Thus, malaria prevention and control strategies addressing communities at high risk of infection should be in place to reduce malaria associated morbidity and mortality in the study localities.

## Data Availability

The data supporting the conclusions conferred in this article is presented in the main paper.
